# Postoperative neurocognitive disorder: migration and crosstalk of inflammation in the peripheral and central nervous system

**DOI:** 10.1186/s13741-025-00640-7

**Published:** 2026-02-14

**Authors:** Xinyi Chen, Wanqiu Yu, Zhaoqiong Zhu

**Affiliations:** 1 Department of Anesthesiology, Affiliated Hospital of Zunyi Medical University, Zunyi, 563003 China; 2 Early Clinical Research Ward, Affiliated Hospital of Zunyi Medical University, Zunyi, 563003 China

**Keywords:** Postoperative neurocognitive disorder, Neuroinflammation, Peripheral inflammation, Blood-brain barrier, Surgery, Anesthesia

## Abstract

Postoperative neurocognitive disorder (PNCD) causeschallenges in addition to that resulting from surgery. These adverse effects not only impact patients in the short term but can also lead to long-term consequences, including an increased mortality rate, a higher risk of developing Alzheimer’s disease, and a decline in post-surgical quality of life and longevity. To better prevent and treat PNCD and improve the prognosis of surgical patients, it is essential to enhance the understanding of the pathogenesis of PNCD. According to recent studies, peripheral inflammation and neuroinflammation may collectively contribute to the development of PNCD. This article discusses the sources of peripheral inflammation, the migration and crosstalk of inflammation between the periphery and the central nervous system, and whether this crosstalk could provide new insights into preventing and treating PNCD.

## Introduction

Recently, the global trend of population aging has intensified, making the health and well-being of the elderly population a growing focus of attention (S. Zhang et al. 2024). With changes in population structure and the development of the healthcare industry, an increasing proportion of elderly patients are undergoing surgery and receiving intraoperative anesthesia, and cognitive changes associated with anesthesia and surgery have garnered increasing attention.

Postoperative brain dysfunction is a common complication in elderly patients following anesthesia and surgery. Among these disorders, Postoperative Neurocognitive Disorder (PNCD), characterized by cognitive impairment that emerges and persists for weeks to months after surgery, is closely associated with adverse patient outcomes. In the long term, patients discharged from hospitals with PNCD are more likely to die within the first three months postoperatively (Monk et al. 2008). For older people, even the occurrence of very mild cognitive impairment may cause them to suffer consequences such as loss of independence and need for high dependence on care measures (Olotu 2020). PNCD is rapidly becoming a significant burden for families and the healthcare system, creating new challenges for an aging society (Yang et al. 2020).

To comprehensively and systematically describe such perioperative cognitive changes, the 2018 consensus of the Nomenclature Consensus Working Group on Perioperative Cognitive Decline recommended to use the term “Perioperative Neurocognitive Disorders (PND)”—which encompasses both preoperative and postoperative cognitive changes—to describe alterations in cognitive function in perioperative patients (Evered et al. 2018). Under this framework, PNCD is defined as a subtype of PND, specifically referring to cognitive decline diagnosed between 30 days and 12 months after surgery. In addition to PNCD, the PND concept also includes other forms of cognitive impairment, such as cognitive decline diagnosed before operation, acute cognitive dysfunction within one week postoperatively or before discharge such as postoperative delirium (POD), and cognitive decline diagnosed within 30 days postoperatively such as delayed neurocognitive recovery (dNCR) (Yu et al. 2024). Although clinical awareness of PNCD is increasing, conclusions from existing studies remain controversial. The pathogenesis of PNCD is complex and not yet fully understood, and there is still a lack of effective prevention and treatment strategies. There is an urgent need to establish and refine relevant intervention approaches.

Inflammation is a dynamic process of the immune system in response to external stimuli. An appropriately scaled inflammatory response aids in pathogen clearance and tissue repair, whereas excessive inflammation may damage multiple organs, including the brain (Margraf et al. 2020). Peripheral inflammation triggers or perpetuates neurodegenerative brain changes, as demonstrated in several past studies (Walker 2018). Animal experiments have convincingly demonstrated that peripheral inflammatory stimuli cause activation of microglia in the brain (Fu et al. 2020), and systemic inflammation induced by peripheral lipopolysaccharide (LPS) injections in mice triggers early astrocyte-responsive neuroinflammation (Guo et al. 2024). Additionally, human studies have indicated that inflammatory cytokines are elevated in cerebrospinal fluid after surgery (Deiner et al. 2020). In developing peripheral inflammation to neuroinflammation, pathways of brain-peripheral communication, such as the blood-brain barrier (BBB), play an important role. Inflammatory signals reaching the central nervous system (CNS) can trigger neuroinflammation that plays a key role in PNCD development, ultimately leading to the development of PNCD (Cheng et al. 2022). In this review, we first briefly introduce PNCD and the potential mechanisms by which neuroinflammation contributes to PNCD development. Subsequently, we explored the possible sources of inflammatory mediators in PNCD as well as the pathways that peripheral inflammatory signals use to reach the CNS and their effects on neurons. Finally, we summarize several drugs and measures that may improve PNCD by controlling peripheral inflammation in the perioperative period.

## Relationship between neuroinflammation and PNCD

There are various hypotheses regarding the occurrence of PNCD, with neuroinflammation considered an important mechanism in the development of many neurodegenerative diseases. Many studies have identified pro-inflammatory signaling molecules in the brains of both patients and animal models with PNCD, demonstrating that neuroinflammation may play a role in this process (Fu et al. 2020). Neuroinflammation is usually defined as an inflammatory response in the CNS. It is triggered by various pathological insults, such as infection, trauma, ischemia, and toxins. Moreover, it is mediated by pro-inflammatory cytokines, including interleukin (IL)−1*β*, IL-6, IL-18, and tumor necrosis factor (TNF) (Leng and Edison 2021). Previous studies have demonstrated that cells in the brain respond to external inflammatory factors and continue to release pro-inflammatory mediators in the brain to promote the onset and progression of neurodegenerative diseases (Heneka et al. 2014). Microglia are central players in neuroinflammation. Under pathological conditions, their overactivation can directly disrupt synaptic plasticity and promote the release of various pro-inflammatory cytokines, including IL-1*β*, thereby triggering subsequent neuroinflammatory responses. IL-1*β* is an important inflammatory factor in neuroinflammation, which significantly impacts the functional maintenance and survival status of neurons. Besides, IL-1*β* induces synaptic loss by increasing the production of prostaglandin E2, leading to presynaptic glutamate release and postsynaptic N-Methyl-D-Aspartate receptor activation (Mishra et al. 2012). TNF is also one of the inflammatory cytokines released by microglia in pathological states. In physiological states, TNF-*α* plays a physiological role in regulating synaptic transmission and plasticity by controlling ionotropic glutamate receptors trafficking. In the pathological state, excessive levels of TNF-*α* exert an inhibitory effect on glutamate transporter proteins, leading to elevated glutamate concentrations in the CNS, which has been associated with the development of various neurological disorders (Olmos and Lladó 2014). All the above findings suggest that neuroinflammation plays a vital role in the pathophysiologic process of PNCD.

## Origin of inflammation in PNCD

The immune system is an indispensable human body component that helps us recognize, reject, and eradicate pathogens and other foreign molecules. The immune system can be divided into two parts: the innate and the adaptive immune systems. The innate immune system responds rapidly to pathogens and provides the body with a direct host defense system; the adaptive immune response develops relatively slowly but can precisely recognize specific antigens (Parkin and Cohen 2001). Surgery can trigger the innate immune system component of the body’s immune system, initiating a systemic inflammatory response (Jia et al. 2023). Using common anesthetics also affects innate and adaptive immunity and has complex interactions with them (Ackerman et al. 2021). Under the blows of anesthesia and surgery, the gut can also produce inflammatory mediators that exacerbate systemic inflammation. Moreover, chronic diseases that may lead to systemic chronic inflammation may be a source of inflammation.

### Surgery

Tissue damage caused by surgery causes localized sterile inflammation at the relevant site due to the release of endogenous damage-associated molecular patterns (DAMPs) by the cells when they are damaged. The DAMPs can be recognized by pattern-recognition receptors, such as toll-like receptors (TLRs) and cytoplasmic nod-like receptors (Huber-Lang et al. 2018; Jia et al. 2023; Yang et al. 2020), inducing the occurrence of sterile inflammation, which is an indispensable link in tissue regeneration and repair (Huang et al. 2024). High mobility group box 1 (HMGB1) is a typical DAMP necrotic cell release during tissue injury. It is a nuclear protein in almost all eukaryotic cells and a secreted protein that can be rapidly released into the circulation after mechanical damage to cells. It can interact with various receptors, such as the receptor for advanced glycation end-products, TLR2 and TLR4, to induce activation of nuclear factor-kappa B (NF-κB) and the production of pro-inflammatory cytokines (Lotze and Tracey 2005).

**Fig. 1:**
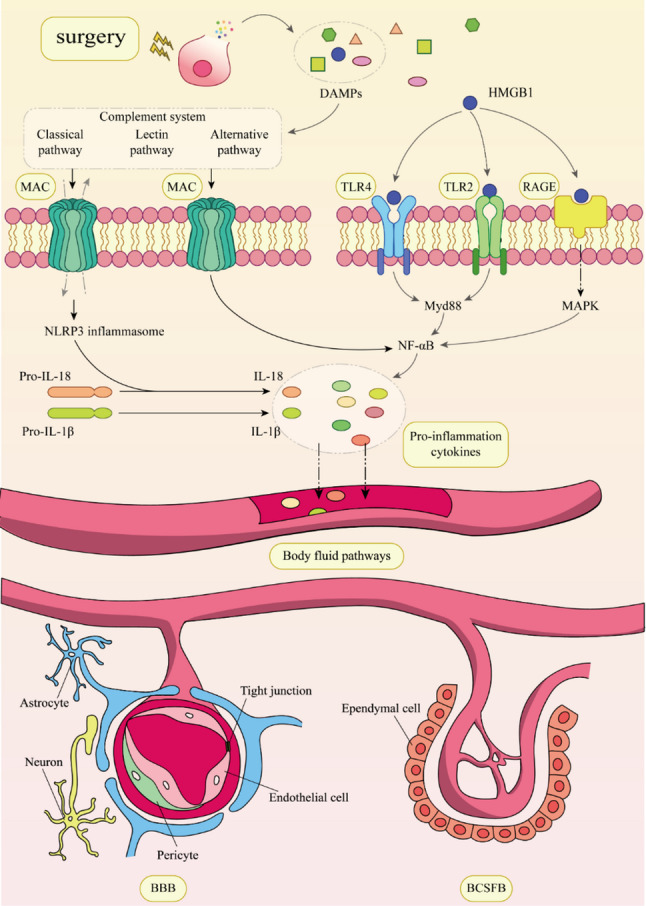
Surgery-induced inflammatory signals are transport through the body’s fluid circulation to the BBB and the blood-cerebrospinal fluid barrier (BCSFB). When tissue cells are damaged by surgery, they release DAMPs. A representative product of DAMPs, HMGB1, can be recognized by cell membrane receptors such as TLR2, TLR4, and receptor for advanced glycation end products(RAGE), which activate NF-κB, leading to the production of pro-inflammatory cytokines. On the other hand, DAMPs can also activate the complement system, through three different pathways, ultimately forms the MAC. The MAC can create pores in the plasma membrane of target cells, allowing small molecules or ions to pass through, thereby activating NLRP3 and resulting in the release of pro-inflammatory cytokines. Additionally, the MAC can directly induce signal transduction without relying on osmotic effects, promoting the expression of pro-inflammatory proteins through NF-κB-dependent transcription. These pro-inflammatory factors can travel through the body’s fluid circulation to reach and disrupt brain barriers such as the BBB and BCSFB, causing dysfunction of these barriers. This allows the inflammatory factors to enter the CNS, triggering neuroinflammation and ultimately leading to the development of PNCD

The complement system is another key contributor to the formation of an inflammatory response, and the same DAMP released by cells after surgical trauma can also activate the complement system. The activated complement cascade can efficiently recognize, mark, and remove debris or DAMP caused by trauma and help initiate regenerative processes (Huber-Lang et al. 2021). The complement cascade consists of over 30 proteins that can be successively activated by three different pathways, namely the classical pathway, the lectin pathway, and the alternative pathway, leading to the formation of the membrane attack complex (MAC) (Ricklin et al. 2010). The MAC may form pores in the plasma membrane on pathogens or target cells leads to osmolysis and activation of the NOD-like receptor family pyrin domain-containing 3 (NLRP3) inflammasomes, releasing pro-inflammatory factors. Without lysis, MAC may induce intracellular signaling and cellular activation. Cells activated by MAC can express pro-inflammatory proteins, assemble inflammatory vesicles, achieve processing, and promote the secretion of IL-1*β* and IL-18, as well as other signaling pathways, through NF-κB-dependent transcription (Xie et al. 2020) (Fig. 1).

### Anesthesia

Propofol, a commonly used intravenous anesthetic in clinical practice, is often employed for the induction and maintenance of anesthesia. For the immune system, propofol has both anti- and pro-inflammatories effects. In anti-inflammatory, it is mainly mediated through its regulation of cytokine biosyntheses such as TNF-*α*, IL-1, IL-6, and IL-10 (Yi et al. 2022), while in pro-inflammatory, it significantly increases IL-5, IL-7, IL-17, and platelet-derived growth factors (PDGF) levels (Kallioinen et al. 2019). Dexmedetomidine is also a commonly used intravenous anesthetic in clinical practice, and it has been suggested that it may promote M2 activity in macrophages through the PPARγ/STAT3 pathway to suppress pro-inflammatory natural immune activation in the liver (Zhou et al. 2020).

Volatile anesthetics play an essential role in clinical anesthesia globally. These volatile compounds may not only affect the immune system of the patient but also that of doctors, nurses, and other personnel, who may suffer from their effects during the perioperative period (Bargellini et al. 2001). Sevoflurane is a clinically common volatile anesthetic, and it exerts anti-inflammatory effects through two main ways. First, it reduces the inflammatory response of the alveolar epithelium (Suter et al. 2007; Yue et al. 2008). Second, it inhibits the production of inflammatory cytokines mediated by the NF-κB and TLR4 pathways (Sun et al. 2015; Watanabe et al. 2013). During the administration of desflurane and sevoflurane, the release of TNF-*α*, IL-8, and IL-1*β* in lung tissue is significantly reduced compared to total intravenous anesthesia with propofol, but the effect on systemic inflammation is negligible (Schilling et al. 2011). Within a few hours after a 2-h sevoflurane general anesthesia in elderly patients who did not undergo surgery, there were few biochemical features of systemic inflammatory activation (Deiner et al. 2020). Several data suggest that, as far as maintenance anesthetics are concerned, the choice of anesthetic agent has little effect on the perioperative peripheral inflammatory response and that individual patient differences and surgical factors may play a more significant role in the inflammatory response (O’Bryan et al. 2022).

Compared to the peripheral system, anesthetics may exert more pronounced direct effects on the CNS. Intravenous anesthetics can enter the brain via the bloodstream and act directly on the CNS. The impact of propofol on cognitive function remains controversial. In infants, young children, and the elderly, propofol may lead to declines in learning and memory. High doses, prolonged administration, or repeated injections may also impair cognitive function. Specific influencing factors are primarily associated with inflammation, imbalances in neurotransmitters, impaired synaptic plasticity, abnormal autophagy levels, and related miRNA molecules (Liu et al. 2022). Furthermore, propofol may contribute to Alzheimer’s disease pathology by influencing amyloid-beta (Abeta) accumulation (Huang et al. 2016). Dexmedetomidine also possesses anti-inflammatory effects in the brain. Studies indicate that it can significantly reduce the risk of dementia in elderly postoperative patients (Sun et al. 2025). Its neuroprotective effects may be attributed to the activation of relevant signaling pathways that reduce the expression of pro-inflammatory factors, inhibit stress responses and cell death, and thereby mitigate neuronal toxicity and ameliorate the occurrence of PNCD by promoting synapse formation and providing neurotrophic support (Zhou et al. 2019).

In contrast to their relatively mild peripheral effects, volatile anesthetics such as sevoflurane and isoflurane may act on the central immune system through various neurotoxic mechanisms, leading to the development of PNCD. Research shows that both sevoflurane and isoflurane can activate microglia, elevate levels of pro-inflammatory cytokines, trigger neuroinflammation, and potentially disrupt blood-brain barrier integrity and neuronal homeostasis (Huang and Zhu 2024; Cho et al. 2024; Miao et al. 2025). Specifically, sevoflurane can induce activation of the NLRP3 inflammasome, leading to hippocampal inflammation and neuronal damage in aged mice. Additionally, sevoflurane may impair cognitive function through mechanisms such as reactive oxygen species(ROS) accumulation and altered expression of ferroptosis-related proteins. Isoflurane, on the other hand, may adversely affect neuronal function by disrupting mitophagy and interfering with glucose metabolism (Yang et al. 2024a; Luo et al. 2025).

### Chronic diseases

As modern medicine significantly extends the human lifespan, the proportion of chronic diseases in all diseases has also risen, and nowadays, chronic diseases are the biggest threat to human health (Kotas and Medzhitov 2015; Yach et al. 2004). Accordingly, chronic diseases are gradually becoming an essential part of the perioperative period that cannot be ignored.

#### Diabetes mellitus

Chronic hyperglycemia in diabetic patients is associated with various complications, including microvascular disease, cardiovascular system disease, and neurodegenerative disease (Weinberg Sibony et al. 2024). Increased oxidative stress (OS) due to hyperglycemia is a major cause of diabetic complications. The OS refers to a disturbance in the prooxidant-antioxidant balance of the cell, resulting in a decrease in the antioxidant capacity of cells (Papachristoforou et al. 2020). The OS can initiate intracellular inflammatory signaling, leading to the expression of pro-inflammatory genes that promote the production of pro-inflammatory cytokines (Bhatt et al. 2020). In addition, OS and inflammation can exacerbate insulin resistance in diabetes, leading to hyperglycemia, while elevated glucose levels further promote the production of inflammatory mediators, creating a vicious cycle that increases the risk of diabetes-related complications, including cognitive decline (Weinberg Sibony et al. 2024).

#### Obesity

Obesity leads to chronic systemic inflammation, which may underlie the association of diseases such as type 2 diabetes and neurocognitive disorders (Rohm et al. 2022). Moreover, obesity can lead to inflammation through various mechanisms. Adipose tissue in obesity is used to express high levels of TNF-*α*, which activates various intracellular signaling molecules and increases the production of inflammatory mediators mediated by the NF-κB pathway (Hotamisligil et al. 1995; Taylor 2021). Circulating free fatty acids produced by adipocyte catabolism can bind to TLR4, inducing the synthesis of inflammatory mediators in macrophages and exacerbating insulin resistance (Muscat et al. 2024). Additionally, obesity can lead to macrophage accumulation in adipose tissue, and activated macrophages can produce inflammatory mediators, including TNF-α and IL-6 (Weisberg et al. 2003), leading to systemic inflammation.

#### Chronic Pain

Chronic pain is defined as pain lasting for 3 to 6 months or more and is now recognized both as a distressing symptom and as a distinct disease entity. Surgery itself and the postoperative inflammatory response can lead to the development of chronic postsurgical or posttraumatic pain (Treede et al. 2019). Among patients with chronic pain, more than half experience comorbid cognitive impairment. Research indicates that chronic pain primarily affects memory processes requiring high levels of attention, such as working memory and the recall of information from long-term memory (Mazza et al. 2018). The underlying mechanism is likely closely related to neuroinflammation mediated by microglial activation. In the state of chronic pain, pro-inflammatory microglia are activated, releasing inflammatory cytokines and chemokines such as IL-6, IL-1*β* and TNF-α, thereby triggering a neuroinflammatory response. This inflammatory response can be observed in multiple brain regions associated with cognition and mood regulation. The sustained activation of microglia not only leads to neuronal dysfunction but may also create a positive feedback loop, further exacerbating the neuroinflammatory process (Cao et al. 2019). This pro-inflammatory state can ultimately lead to synaptic remodeling, alterations in brain connectivity and neural network function, resulting in impaired cognitive function (Inoue and Tsuda 2018). Numerous animal studies have shown that chronic pain increases pro-inflammatory activation of microglia and neuroinflammation in the brain. In animal models of different types of chronic pain, activation of microglia has been detected in cognition-related brain regions such as the prefrontal cortex (Xu et al. 2017) and the hippocampus (Ji et al. 2018; Dai et al. 2019). Furthermore, neuropathic, inflammatory, and idiopathic chronic pain have all been demonstrated to be associated with cognitive dysfunction.

### Gut

The gut is also a source of peripheral inflammatory factors. The gastrointestinal tract is the largest digestive and immune organ in the human body. Recently, with the discovery of the brain-gut axis, the intestinal flora has also become an important topic in the study of CNS disorders. Many studies have demonstrated that gut microbiota plays a vital role in maintaining immune, metabolic, and tissue homeostasis in the gut and the CNS (Carloni and Rescigno 2023). The significant gut microorganisms are colonized in the mucus layer formed by mucins secreted by goblet cells (Gustafsson and Johansson 2022). Under physiological conditions, the gut microbiota is well balanced with the host intestinal mucosal immune barrier. Moreover, the well-balanced gut microbiota is disrupted when the intestinal microbiota is dysregulated by various factors, resulting in intestinal barrier dysfunction. The TLR expressed in the gut is crucial in maintaining intestinal homeostasis and improving intestinal barrier integrity. Besides, it can be activated by factors such as LPS in the cell wall of gram-negative bacteria and DAMPs such as HMGB1 (Poltorak et al. 2000; Zhang et al. 2023). After surgery and anesthesia, the abundance of the intestinal microbiota changes, increasing the proportion of gram-negative bacteria (Lederer et al. 2017). The LPS from the cell wall of gram-negative bacteria activates TLR4 on the surface of intestinal epithelial cells, leading to increased intestinal barrier permeability (Guo et al. 2013; X.-Q. Wang et al. 2021). Sequentially, this allows the entry of intestinal bacteria and toxic metabolites into the circulation of the intestinal tract, ultimately resulting in the onset of systemic inflammatory responses (Lu et al. 2022).

In addition to disrupting the intestinal barrier, dysbiosis of the gut microbiota can also influence brain metabolism and vascular function through disrupted microbial metabolites, thereby contributing to the occurrence of PNCD. Bacterial fermentation-derived short-chain fatty acids (SCFAs) can enter the brain and regulate microglial function, potentially triggering neuroinflammation (Erny et al. 2015; Yang et al. 2024b). Tryptophan metabolites of gut origin may suppress CNS inflammation by activating the aryl hydrocarbon receptor (AhR) signaling pathway in astrocytes (Rothhammer et al. 2016). Bile acids can cross the blood-brain barrier and bind to the farnesoid X receptor (FXR) and Takeda G protein-coupled receptor 5 (TGR5) on neurons and glial cells in the CNS, exerting protective effects in conditions such as Parkinson’s disease, subarachnoid hemorrhage, and middle cerebral artery occlusion (Grant & DeMorrow 2020; Wu et al. 2024).

The gut microbiota is highly sensitive to environmental changes. Stress during surgical anesthesia, gastrointestinal motility abnormalities induced by anesthetic drugs or muscle relaxants, and perioperative antibiotic use may all increase the risk of intestinal dysbiosis (Sun et al. 2023). It has been demonstrated that the abnormal gut microbiota induced by surgery or anesthesia is age-dependent, with a significant decrease in microbiota abundance with increasing age (Liufu et al. 2020). These factors may lead to gut microbiota dysbiosis, resulting in impairment of the intestinal barrier function and dysregulation of associated metabolites, which can directly or indirectly contribute to neuroinflammation.

## Pathways of peripheral inflammatory signals to the CNS

A finding confirmed in previous studies demonstrates that combining surgical trauma and anesthetic injury leads to an inflammatory response in vivo that results in neuroinflammation (Kapila et al. 2014). It has been reported that surgical trauma can shift the dynamic balance of microglial activation toward M1 polarization in aged mice, exacerbating neuroinflammation and leading to cognitive impairment (Zhang et al. 2020). This demonstrates that the CNS is not an “immune-privileged” compartment unaffected by and unresponsive to peripheral inflammation (Engelhardt et al. 2017). Many studies have indicated that systemic inflammatory mediators can signal the brain through two different pathways: neural and humoral (J. Xie et al. 2022a).

### Body fluid pathways

Humoral signals circulate in the body along the blood system, including inflammatory factors and immune cells. Under physiological conditions, the brain is protected by a brain barrier through which only soluble lipid molecules of low molecular weight (below 0.4–0.6 kDa) and positively charged can pass (Bellettato and Scarpa 2018). Whereas, during systemic inflammation, inflammatory factors may disrupt the integrity of the brain barrier, allowing peripheral molecules and immune cells to infiltrate the CNS. Furthermore, the circumventricular organs (CVOs), which lack the typical barrier structure, may serve as a pathway for humoral signals, such as inflammatory factors, into the CNS.

#### Systemic inflammation impacts the CNS barrier and CVOs

The function of the CNS is maintained by the highly coordinated activity of many cell types within the neurovascular unit (NVU), including vascular cells (endothelial cells (ECs), pericytes, and smooth muscle cells), glial cells (astrocytes, oligodendrocytes, and microglial cells) and neurons (Zlokovic 2011). The BBB is centrally located in the NVU and is formed by brain ECs in the capillary wall, astrocyte endfeet, and pericytes embedded in the basement membrane of capillaries (Sweeney et al. 2019). The BBB separates the CNS from the peripheral circulation, which is an important part of protecting neurons from the various factors in the somatic circulation and maintaining the highly regulated internal environment of the CNS, which is essential for maintaining normal synaptic and neuronal structure. After BBB destruction, various toxins and pathogens from the somatic circulation enter the brain, triggering neuroinflammation and neurodegeneration, which can lead to various neurological diseases (Obermeier et al. 2013; Sweeney et al. 2018).

##### ECs

The ECs are a core component of the BBB with specific inward transport proteins such as glucose transport 1 and outward transport proteins such as p-glycoprotein (P-gp) (Zlokovic 2008). As a major barrier component, EC is an important target for the attack of peripheral inflammatory factors such as LPS, TNF-α, and IL-1*β* (Chen et al. 2024). Among them, LPS may impair P-gp activity by activating the mitogen-activated protein kinase (MAPK) signaling pathway and increasing the expression of NF-κB in activated B-cells within ECs, which in turn induces endoplasmic reticulum stress and mitochondrial damage in ECs, ultimately leading to cell apoptosis. (Bernhart et al. 2018; Cardoso et al. 2012; Quan et al. 2003). Activation of the NF-κB signaling pathway induces the transcription of various pro-inflammatory genes, including cytokines, chemokines, and adhesion molecules (such as intercellular adhesion molecule-1 (ICAM-1) and vascular cell adhesion molecule-1 (VCAM-1). This leads to the degradation of proteins in ECs and tight junctions (TJs), resulting in reduced barrier impermeability, while also enhancing leukocyte adhesion and migration. Such structural disruption promotes the entry of plasma molecules and immune cells into the brain parenchyma, thereby contributing to a neuroinflammatory state (Wettschureck et al. 2019; Ponce-Lopez 2025). Furthermore, The IL-1 receptors are expressed on ECs, which are indispensable for their mediation of disease behavior. They drive leukocyte recruitment to the CNS to damage neurons and induce microglia to produce IL-1 in the brain following IL-1 stimulation of ECs (Liu et al. 2019).

##### Tight junctions

The TJs are unique barriers between ECs and consist of a complex of three molecules: occludins, claudins, and junction adhesion molecules (Cong and Kong 2020; Galea 2021). Claudins and occludins are TJ’s main protein components, which are connected to the cytoskeleton through members of the zonulin family (Wang et al. 2021). Among claudins, claudin 5 is highly expressed in brain microvascular endothelial cells (BMEC) and participates in constituting the backbone of the TJ chain to regulate BBB permeability (Yang et al. 2021). In vitro BBB modeling has indicated that claudin 5 is a common target of attack by inflammatory mediators such as IL-1*β* and TNF-*α* (Aveleira et al. 2010; Beard et al. 2014), and there is a direct relationship between claudin 5 dysregulation and BBB dysfunction (Greene et al. 2022). Additionally, the inflammatory cytokines IL-1*β* and IL-8 are associated with the loss of the proteins occludin and zonula occludens (ZO)−1 (Kim et al. 2022).

##### Pericytes

Pericytes are cells that cover the CNS capillaries and play a key role in integrating endothelial and astrocyte functions in the NVU and regulating the BBB (Armulik et al. 2010). With stimulated by inflammatory factors such as IL-1*β*, TNF-*α*, and LPS, and pericytes secrete many functioning chemokines to attract circulating leukocytes to the brain through a concentration-directed gradient (Armulik et al. 2011; Guijarro-Muñoz et al. 2014; Pieper et al. 2014). Immunologically activated pericytes also secrete various pro-inflammatory mediators, including IL-1*β*, TNF-*α*, and IL-6, which induce a pro-inflammatory state in astrocytes, microglia, and ECs, thereby promoting apoptotic neuronal death (Rustenhoven et al. 2017).

##### Blood-cerebrospinal fluid barrier

The blood-cerebrospinal fluid barrier (BCSFB) consists of choroid plexus (CP) epithelial cells located in the ventricles of the brain (Mapunda et al. 2022), and similar to the BBB, the CP epithelial cells have distinctive apical TJs between them (Santos et al. 2017). The BCSFB forms a protective barrier between the blood and the cerebrospinal fluid within the ventricles. It plays a crucial role in safeguarding the brain by restricting the passage of substances into the brain tissue (Ghersi-Egea et al. 2018). In addition to their barrier function, CP epithelial cells have the function of secreting cerebrospinal fluid (Engelhardt and Sorokin 2009). Under the influence of inflammation, the integrity of the BCSFB is compromised and may lead to infiltration of cells of the immune system, which can contribute to further development of neuroinflammation (Mineiro et al. 2024). Studies have indicated that systemic injection of LPS, which induces peripheral inflammation, can lead to upregulation of TLR4 in CP cells, potentially resulting in decreased occludin expression in the BCSFB (Chakravarty and Herkenham 2005; Laflamme and Rivest 2001; Quan et al. 1997). In addition, activation of NF-κB upregulates matrix metalloproteinase 9 production and induces claudin 5 degradation in BCSFB, which may also lead to alterations in the BCSFB barrier (Chiu and Lai 2013). In contrast, in sepsis, CP epithelial cells sense peripheral inflammation and transmit information about peripheral inflammation to the CNS by releasing extracellular vesicles into the cerebrospinal fluid (Balusu et al. 2016).

##### CVOs

The CVOs are highly vascularized regions surrounding the third and fourth ventricles that lack a BBB, and due to their characteristics of not expressing TJ proteins and allowing passage of molecules of blood origin with a molecular weight of < 10,000 (Miyata 2022), CVOs are a significant entry pathway for blood-derived inflammatory cytokines and pathogens, providing a specialized niche of initiating rapid and early neuroinflammatory responses in the brain (Kawai et al. 2020; Sisó et al. 2010). The CVOs can be categorized according to their primary functional function into sensory CVOs, including the organum vasculosum of the lamina terminalis, subfornical organ, and area postrema, and secretory CVOs, which encompass the neurohypophysis, median eminence, pineal gland, and intermediate lobe of the pituitary gland (Cottrell and Ferguson 2004). Sensory CVOs are conduits for circulating cytokines and chemokines, DAMPs, and pathogen-associated molecular patterns (PAMPs) into the brain (Bourhy et al. 2022). The CVO can express pattern recognition receptors, including TLR2 and TLR4 (Chakravarty and Herkenham 2005; Murayama et al. 2019), and cytokine receptors, such as IL-1 and TNF-α1 (Korim et al. 2019; Wei et al. 2013). Following stimulation by peripheral inflammatory stimuli, the transcription of genes encoding pro-inflammatory molecules and activating inflammatory signaling molecules occur more rapidly in CVO than in other brain regions (Kawai et al. 2020). Moreover, microglia and macrophages in the CVO consistently exhibit an activated state and express M1/M2 marker proteins. After administering LPS stimulation to mice, the expression of complement protein C1q in microglia within the CVOs and their adjacent brain regions significantly increases, possibly related to the early physiological response to neuroinflammation (Kawai et al. 2020). All these studies suggest that CVOs are important pathways for peripheral immune signals to enter the CNS (Zhang et al., 2024).

#### Intrinsic CNS cellular effects

##### Astrocytes

Astrocytes are the most abundant neuroglia in the brain, accounting for approximately 25% of the brain volume (Tower and Young 1973). They have various functions, such as recycling neurotransmitters, regulating metabolic homeostasis, providing nutrition to neurons, composing the BBB, and participating in immune responses (Lee et al. 2022). They play a crucial role in maintaining the integrity of the BBB and regulating its function (Huang et al. 2021). Under inflammatory conditions, astrocytes secrete vascular endothelial growth factor A (VEGF-A), downregulating claudin 5 and occludin expression on ECs, making it easier for peripheral lymphocytes to enter the CNS and causing neuroinflammation (Argaw et al. 2012; Schumacher et al. 2024). The C-C chemokine receptor 5 (CCR5) is the primary receptor for C-C motif chemokine ligand 5 (CCL5), and the CCL5/CCR5 axis, which consists of the two together, plays a vital role in neuroinflammation (Ma et al. 2023). Studies have indicated that severe peripheral inflammation increases the expression of CCR5 in astrocytes, leading to a decrease in expressing tight junction proteins, such as ZO-1 and claudin 5, ultimately disrupting BBB integrity (Lin et al. 2023).

##### Microglia

Microglia are resident immune cells within the CNS, accounting for approximately 10% of all cells in the adult human CNS (Masuda et al. 2020). As mentioned above, they are not only involved in the development and maintenance of neuroinflammation, but microglia in their activated state also directly impact the functional integrity of the BBB (Ronaldson and Davis 2020).

Based on their functions, microglia can be classified into two opposing types: M1 and M2 phenotypes, and microglia can shift between these two different phenotypes (Guo et al. 2022). The M1 phenotype is the pro-inflammatory phenotype of microglia, which is capable of producing various pro-inflammatory cytokines, including IL-1*β*(Luo et al. 2021). The M1 phenotype of microglia is mediated by TLR 4, interferon-γ (IFN-γ) receptor, or granulocyte-macrophage colony-stimulating factor (GM-CSF) receptor (Ponomarev et al. 2007; Moritz et al. 2017; Yang et al. 2018a). It can lead to increased production of pro-inflammatory cytokines and chemokines such as TNF-*α*, IL-1*β*, and IL-6 (Smith et al. 2012; Subramaniam and Federoff 2017), as well as enhanced expression of cyclooxygenase-2 (COX-2) and inducible nitric oxide synthase (iNOS)(Yang and Rosenberg 2011), increased inflammation and OS, ultimately leading to dysfunction of the BBB. Furthermore, increased synaptic phagocytosis by microglia can also impair the BBB, leading to more severe inflammatory responses(Merlini et al. 2019). The M2 phenotype of microglia is mediated by IL-4, Fcgamma receptors, and IL-10 receptors (Subramaniam and Federoff 2017). After transformation, these microglia can secrete anti-inflammatory mediators such as IL-10 and TGF-*β*1 and protect the BBB in neurological diseases (Ronaldson et al. 2009; Wang et al. 2016; Ronaldson and Davis 2020).

Microglia play a dual role in maintaining the integrity of the BBB. In the early stages of inflammation, due to the release of the chemokine CCL5 by ECs, microglia migrate toward the brain vessels, aggregate, and come into contact with ECs, and assemble TJs by providing claudin 5 directly to ECs to maintain BBB integrity. However, as inflammation progresses, microglia adopt a phagocytic phenotype, engulfing astrocyte endfeet and impairing BBB function. This disruption leads to the leakage of systemic material into the parenchyma, causing widespread neuroinflammation (Haruwaka et al. 2019).

In addition, In the M1 phenotype, microglia can directly disrupt synaptic plasticity, leading to learning and memory deficits in PNCD (Saxena et al. 2021). Synaptic plasticity is essential for maintaining normal brain function. In neurodegenerative contexts, aberrant microglial activation and phagocytic activity are closely associated with synaptic loss and cognitive dysfunction (Li et al. 2025). Under physiological conditions, the phagocytic capacity of microglia helps clear neuronal and synaptic debris. However, under pathological conditions, excessively active microglial phagocytosis can disrupt normal synaptic structures and lead to impaired neural network function (Wang et al. 2020). Complement activation can also mediate microglia-dependent synaptic engulfment. The accumulation of complement components regulates interactions between microglia and synapses in the hippocampus, which may further contribute to declines in memory function (Krukowski et al. 2018). Together, these mechanisms contribute to synaptic plasticity dysfunction.

With advances in single-cell transcriptomics, researchers have gained new insights into the heterogeneity and plasticity of microglia (Masuda et al. 2020). It is now understood that microglia are not limited to two opposing and fixed phenotypes but comprise multiple distinct subpopulations beyond the classical M1/M2 dichotomy(Wu et al. 2025). Microglia continuously sense and respond to their microenvironment, adapting to injury, infection, or neurodegeneration by transitioning into various subtypes. In Alzheimer’s disease research, a unique microglial cluster with a distinct gene expression profile has been identified: disease-associated microglia (DAM). These cells play a protective role by clustering around amyloid plaques, forming a barrier that may limit plaque expansion through phagocytosis and clearance of Abeta, thereby suppressing neuroinflammation(Keren-Shaul et al. 2017). More recently, additional microglial categories have been identified based on gene expression signatures, such as aging-dependent microglia (ADEM), microglial neurodegenerative phenotype (MGnD), and MS-inflammatory microglia (MIMS) (Krasemann et al. 2017; van der Poel et al. 2019; Li et al. 2023; Wang et al. 2025).

##### T Cells

Thymus-derived T cells are key players in antigen-specific immune responses. Due to the “immune-privileged” status of the brain, T cells are relatively scarce in the healthy CNS (Ellwardt et al. 2016). However, following cerebral insult, T cells—particularly CD4⁺ T cells—can infiltrate the brain parenchyma and differentiate into various subsets (such as Th cells and Tregs) to modulate inflammation and coordinate tissue repair (Zhang et al. 2021). T cells are broadly divided into three functional subtypes: CD8⁺ cytotoxic T cells, CD4⁺ helper T (Th) cells, and CD4⁺ regulatory T (Treg) cells. Each subset plays a distinct role in neuroinflammation. CD4⁺ Th cells can further differentiate into subtypes including Th1, Th2, Th17, and other specialized phenotypes (Li et al. 2025a). Surgery-induced cognitive impairment in mice is associated with elevated IL-17 (primarily produced by Th17 cells) and reduced IL-10 (mainly secreted by Tregs) (Tian et al. 2015). Th1 and Th17 cells promote neuroinflammation; upon activation by inflammatory signals, they release cytokines such as IFN-γ, TNF-α, and IL-17, thereby amplifying neuroinflammatory responses and disrupting blood-brain barrier integrity (Li et al. 2025a). In contrast, Treg cells exert anti-inflammatory and reparative functions and help prevent excessive neuroinflammation. Studies suggest that Treg dysfunction may contribute to surgery-related cognitive decline in aged mice. Blocking CD25 signaling has been shown to restore connexin expression, preserve blood-brain barrier function, reduce hippocampal inflammation, and rescue cognitive performance in these animals (Zhou et al. 2023).

### Neural pathways

#### The vagus nerve

In the nervous system, the vagus nerve plays a crucial role in regulating inflammatory homeostasis by transmitting reflex signals (Andersson and Tracey 2012). The vagus nerve originates directly from the brain and innervates the corresponding organs through motor and sensory nerve fibers (Pereira and Leite 2016). Its afferent nerves are in an excellent position to detect immune-related events in the periphery and generate appropriate autonomic, endocrine, and behavioral responses through central reflex pathways that can transmit various signals from the periphery to the brain, including signals from peripheral inflammatory processes (Berthoud and Neuhuber 2000; Jia et al. 2023). It has been indicated that severing the vagus nerve can block signaling pathways between the periphery and the brain, attenuating LPS-induced central disease (Dilger and Johnson 2008). When the body’s inflammatory response is activated by DAMP or PAMP, these signals are transmitted by the vagus nerve to the nucleus tractus solitarius in the brainstem, which activates microglia, leading to neuroinflammation. It has also been demonstrated that peripheral inflammation can increase levels of glutamate and glutamate receptor subunit NR 2B in the brain, activating brain mast cells and amplifying neuroinflammation (Yang et al. 2022). The solitary tract nucleus further activates efferent motor signals in the neighboring dorsal motor nucleus of the vagus (DMV) (Andersson and Tracey 2024). Interestingly, this signal is transmitted down to the peripheral system, which in turn prevents excessive inflammation, tissue damage, and death in the organism (Kressel et al. 2020). This vagal stimulation leads to a systemic reduction in producing inflammatory cytokines and has been termed the “cholinergic anti-inflammatory pathway” (Mannon et al. 2019).

The vagus nerve is also a crucial component of the brain-gut axis. Vagus afferent fibers are distributed throughout all layers of the digestive tract wall except the epithelial layer and avoid direct contact with the gut microbiota (Wang and Powley 2007). Consequently, vagus nerve fibers in the gut sense signals from the gut microbiota by sensing bacterial compounds and metabolites diffusing into the epithelial layer or through other cells transmitting luminal signals (Bonaz et al. 2018). Similar to the vagus nerve located in other parts of the body, the vagus nerve fibers in the intestine transmit the sensed signals to the solitary tract nucleus located in the brainstem (Agirman et al. 2021), activating microglia and leading to the occurrence of neuroinflammation (J. Xie et al. 2022a). Similarly, the CNS can regulate intestinal inflammation through the descending vagus nerve pathway using the DMV. The cholinergic efferent vagus nerve fibers can stimulate intestinal neurons, inhibiting the release of inflammatory cytokines, including IL-1*β*, IL-6, IL-18, and TNF-*α* by macrophages (Bonaz et al. 2018). Inflammation-mediated vagal afferent fibers also activate the hypothalamus-pituitary-adrenal (HPA) axis. The HPA axis is an important part of the gut-brain axis, which is involved in and controls the stress response and regulates many physical activities. When the body undergoes a stress response, the paraventricular nucleus of the hypothalamus synthesizes and releases corticotropin-releasing hormone, which in turn stimulates corticotropic cells in the anterior pituitary gland to release adrenocorticotropic hormone (ACTH). The ACTH stimulates the release of the end-product cortisol from the nucleus tractus solitarius of the adrenal cortex (Rusch et al. 2023; X.-Q. Wang et al. 2021c). Cortisol receptors are present in various organs in the body, and the brain is the key target organ for glucocorticoids (GCs). Dysfunction of the HPA axis can lead to neurodegeneration, adversely affecting cognitive function (Ouanes and Popp 2019).

## Therapeutic directions for controlling peripheral inflammation in PNCD

The above evidence suggests that peripheral inflammation is involved in developing PNCD. Measures to suppress peripheral inflammation may help to prevent or ameliorate PNCD. This includes optimizing perioperative management plans for patients and using anti-inflammatory drugs to combat PNCD from a multidisciplinary and multifaceted approach to maximize the benefits for patients.

### Optimizing perioperative management

**Fig. 2:**
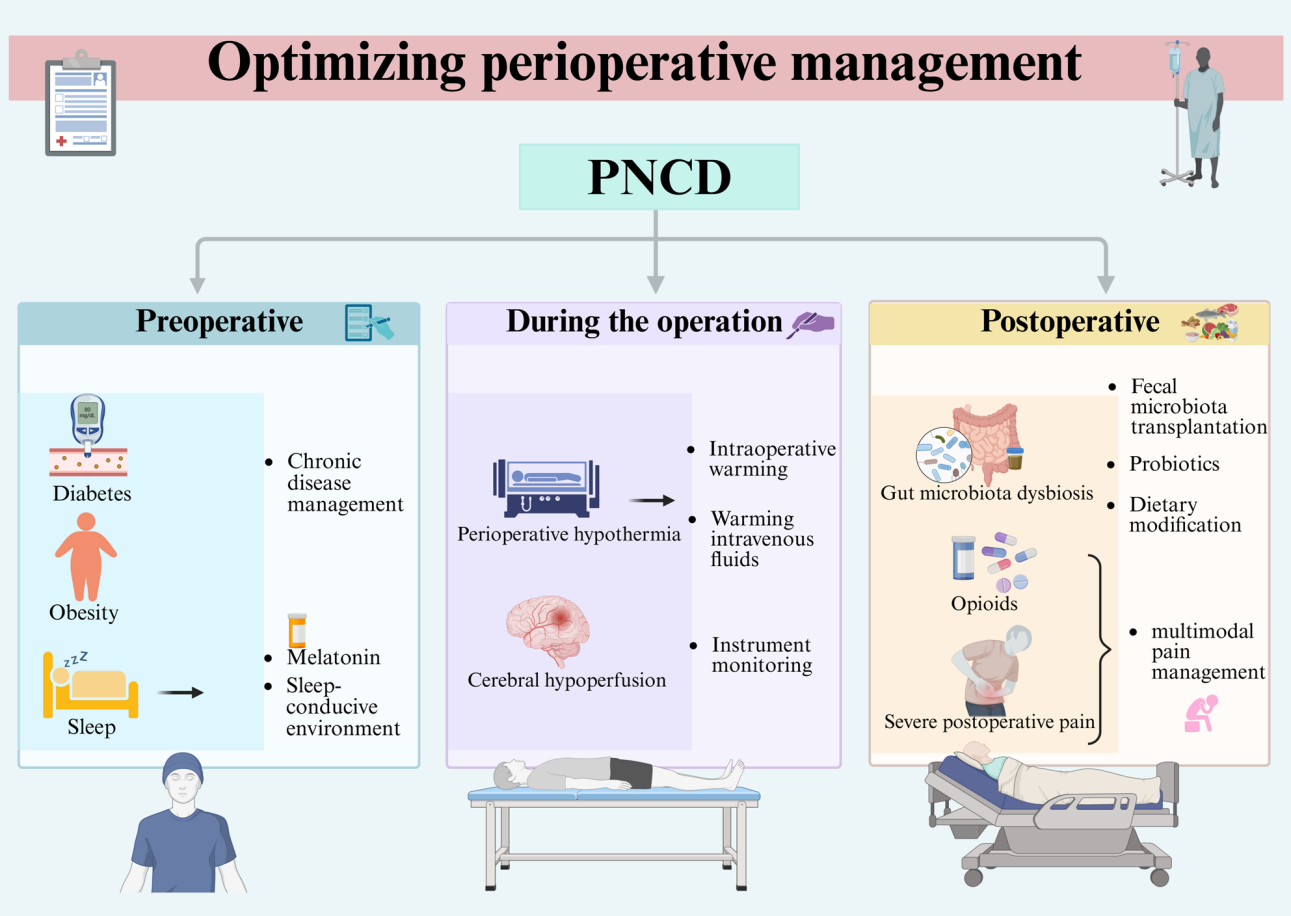
Optimizing perioperative management. Preoperative chronic disease management should be done for people with diabetes, obesity and other chronic diseases, and measures should be taken to improve sleep in patients with preoperative sleep disorders. Intraoperative insulation and cerebral oximetry should be done for patients to prevent intraoperative hypothermia and inadequate cerebral perfusion. Postoperatively, the gut microbiota should be actively improved, and multimodal pain management strategies should be applied to reduce patients' pain and opioid use

Identifying risk factors for patients with PNCD in the early preoperative period and optimizing their preoperative physical status can reduce the risk of PNCD (Hasan et al. 2020) (Fig. 2). As mentioned above, chronic diseases such as obesity and diabetes, present in the preoperative period, are among the risk factors for PNCD. Therefore, managing chronic diseases in the preoperative period improves inflammation production and reduces the probability of PNCD (Needham et al. 2017). Sleep is crucial for brain health and cognitive function, and perioperative sleep disturbances are prevalent in surgical patients (O’Gara et al. 2021; Wanget al. 2021). Several data suggest that sleep disturbances present preoperatively may be associated with postoperative delirium (Fadayomi et al. 2018). Sleep restriction may damage the integrity of the BBB (He et al. 2014), allowing peripheral inflammatory factors to enter the brain. Sleep also affects inflammatory mediators, leading to chronic, systemic low-grade inflammation, and has been associated with various diseases affected by systemic inflammation, such as diabetes and neurodegeneration (Besedovsky et al. 2019). Sleep-promoting medications such as melatonin and environmental modifications, including earplugs, eye masks, and soothing music, can facilitate sleep, maintain perioperative circadian rhythms, and reduce the risk of PNCD (Wang et al. 2021). Besides, developing anesthesia and surgical plans that are more suitable for patients, avoiding the use of neuromodulatory medications, including benzodiazepines and anticholinergics, and reducing the overall dosage of opioids can have a positive effect on preventing PNCD (Safavynia et al. 2022).

Specific intraoperative interventions can also reduce the risk of patients developing PNCD. During surgery, three factors may cause patients to develop perioperative hypothermia (a core temperature drop to < 36 ℃) (Campbell et al. 2015). These factors are: prolonged exposure of the surgical incision, disrupted normal thermoregulation caused by anesthetic medications, and administration of large volumes of intravenous and irrigation fluids. Perioperative hypothermia can lead to prolonged effects of anesthetics, impaired immune function, poor wound healing, and increased risk of infection (Sessler 1997; Torossian et al. 2015). It has been indicated that even mild intraoperative hypothermia is significantly associated with an increased risk of POD (Ju et al. 2023). Actively warming patients and using warmed intravenous infusions during surgery can effectively prevent the occurrence of hypothermia (Campbell et al. 2015; Torossian et al. 2015). Additionally, ensuring adequate oxygen supply to patients during surgery is vital for oxidative phosphorylation and adenosine triphosphate production in the body, and overall or localized inadequate cerebral perfusion has been associated with PNCD development (Safavynia et al. 2022). It has been demonstrated that using near-infrared spectroscopy technology for cerebral oximetry-guided interventions during cardiac surgery to improve intraoperative cerebral coaptation reduces the incidence of PNCD (Uysal et al. 2020).

Adopting an effective preventive program after surgery can improve patients’ prognosis and reduce the probability of cognitive impairment. Severe postoperative pain is associated with the development of delirium, and opioids, which are commonly used as clinical analgesics, play a significant role in the analgesia of acute pain arising from trauma or pain (O’Gara et al. 2021). However, opioid use may contribute to the increased incidence of PNCD (Awada et al. 2019). Reducing opioid use and utilizing a multimodal pain management strategy that combines opioid and non-opioid medications may improve the occurrence of cognitive impairment (Wilson et al. 2022).

Surgery, anesthesia, and opioid use may disrupt the gut microecology, leading to ecological imbalances that can induce PNCD. Implementing appropriate interventions to minimize damage to the microbiota ecosystem and repair microecological dysbiosis may be promising therapeutic strategies (Huang et al. 2025). Fecal microbiota transplantation is a novel approach for treating gut dysbiosis, which can be performed by transplanting the microbiota of an ideal donor to replace the gut microbiota of the target recipient, thereby improving the cognitive function of the patient (DuPont et al. 2023; Sun et al. 2023). Probiotics can promote the growth and reproduction of beneficial intestinal flora and improve the balance of intestinal flora, thereby reducing cognitive impairment after surgery (Sun et al. 2023; X.-D. Yang et al. 2018b). Dietary modification is also an essential part of the therapeutic strategy, and a diet high in dietary fiber is beneficial for maintaining the diversity of the gut microbiota and the suppression of inflammation. Additionally, this study has indicated that dietary restriction can potentially ameliorate anesthesia- and surgery-induced cognitive dysfunction by improving the regulation of the intestinal microbiota and suppressing inflammatory responses in hippocampal microglial cells (Ren et al. 2024). 

### Drug therapy

Non-steroidal anti-inflammatory drugs (NSAIDs) are commonly used anti-inflammatory drugs in clinical practice, which function primarily by inhibiting COX-1 and COX-2, which are involved in the synthesis of prostaglandins, resulting in analgesic, anti-inflammatory, and antipyretic effects (Day and Graham 2013). The NSAIDs ibuprofen was indicated to inhibit LPS-induced cognitive dysfunction and neuroinflammation in rats (Alagan et al. 2019), which may be attributed to the fact that ibuprofen inhibits the production of pro-inflammatory cytokines and the activation of microglia and reactive astrocytes by weakening stress signaling pathways (Huang et al. 2018). A single injection of ibuprofen before abdominal surgery in rats improved postoperative spatial memory (Oberman et al. 2021). Parecoxib, an injectable prodrug of the selective COX-2 inhibitor vadixib (Cheer and Goa 2001), significantly reduced the incidence of PNCD and improved Mini-Mental State Examination (MMSE) score when administered intravenously in the perioperative period (Huang et al. 2020).

The GCs are commonly used immunosuppressive agents in clinical practice for treating various inflammatory diseases, including asthma, allergies, and septic shock (Dendoncker and Libert 2017). The synthetic GC dexamethasone, which is routinely used in anesthesia practice, is considered one of the ideal perioperative medications (Bansal et al. 2024) and can prevent postoperative nausea, vomiting, and airway obstruction occurring after extubation (Safavynia et al. 2022). It has been indicated that preoperative application of dexamethasone reduces the inflammatory response after cardiac surgery, thereby reducing the risk of early PNCD (Glumac et al. 2017). A single intrathecal injection of dexamethasone and levobupivacaine resulted in a significant reduction in the rate of complications such as delirium and PNCD (Sakic et al. 2023). While other studies yielded opposite results, a study on cardiac surgery revealed that a high dose of dexamethasone during cardiac surgery did not reduce the risk of developing PNCD after surgery (Ottens et al. 2014). It has also been observed that dexamethasone significantly reduced severity scores in patients who had developed POD symptoms; however, there was a non-significant difference in the incidence of POD (Kluger et al. 2021). Recently, it has been found that the use of perioperative GCs may not reduce the incidence of postoperative PNCD (Xie et al. 2022b). In conclusion, there remains a controversy about whether GCs are effective for PNCD, and further studies are needed to prove it.

Traditional Chinese Medicine therapiesmedicine may also prevent the development of PNCD by reducing the inflammatory response caused by surgery. Shenmai and Shenfu injections are two ginseng containing preparations based on traditional Chinese herbs. A previous study found that preoperative intravenous administration of Shenmai injection and postoperative intravenous administration of Shenfu injection can promote the recovery of consciousness in aged rats after surgery and prevent the occurrence of PNCD. In a mouse model of middle cerebral artery occlusion and reperfusion (MCAO/R), Shenfu Injection was shown to downregulate the expression of the RAGE, activate the PI3K/Akt pathway, inhibit NF-κB activation after cerebral ischemia, and attenuate neuroinflammation (Li et al. 2025d). A randomized clinical trial involving patients with myocardial infarction further demonstrated the safety of Shenfu Injection in the context of ischemia-reperfusion injury (Wang et al. 2021b). Additionally, acupuncture—a common Traditional Chinese medicine treatment—may offer adjunct benefits in managing PNCD. Electroacupuncture (EA), a contemporary adaptation of traditional acupuncture, has been shown in some studies to inhibit neuroinflammation and improve neurological function after ischemic brain injury, partly through suppressing ferroptosis (Li and Li 2025). It has also been studied in the context of improving awakening quality after general anesthesia (Bu et al. 2024).

Beyond Chinese medicine, curcumin—a compound derived from turmeric with origins in Indian traditional medicine—represents a promising multi-target agent against neurodegenerative disorders. Modern research indicates that curcumin attenuates oxidative stress and suppresses key inflammatory mediators including NF-κB, COX-2, and iNOS. It may also confer neuroprotection by modulating cell death pathways, inhibiting amyloid-*β* aggregation, and enhancing mitophagy (Lehoczki et al. 2025). Similarly, saffron—used in traditional Persian medicine for treating erysipelas—has been investigated in modern contexts for its potential therapeutic effects in neurodegenerative diseases such as Alzheimer’s and Parkinson’s (Tufail et al. 2025). These traditional therapeutic approaches broaden the perspective on PNCD treatment. However, since traditional medicines are often complex mixtures with challenges in standardization and quality control, further research and exploration are still needed to fully elucidate their efficacy and mechanisms.

## Concluding remarks

Peripheral inflammation is important in developing PNCD and is closely related to CNS inflammation. After the occurrence of peripheral inflammation caused by surgery and other factors, inflammatory mediators can directly enter or transmit inflammatory signals into the brain through two different pathways: nerve and body fluids, inducing neuroinflammation, which ultimately leads to pathologic changes in the structure or function of the brain, and triggers many neurodegenerative diseases, including PNCD. Given this strong link between peripheral and neuroinflammation, modulation of the peripheral inflammatory response may be effective in preventing and treating PNCD. Clinically used NSAIDs, GCs, and extracts of some Chinese medicines may play a role in reducing peripheral inflammation in this context. Additionally, developing patient-specific PNCD prevention strategies in the perioperative period and interventions in the early stages can help reduce the incidence of PNCD. However, regarding the choice of perioperative anesthetic regimen, although some studies have indicated that some anesthetics may induce the development of peripheral inflammation, there remains a controversy over whether the choice of anesthetic agent during surgery is an independent risk factor for postoperative neurocognitive impairment, and further studies are needed to prove it.

## Data Availability

No datasets were generated or analysed during the current study.
